# Supplementary material for: LinRace: cell division history reconstruction of single cells using paired lineage barcode and gene expression data
Source: Nat Commun. 2023 Dec 16;14:8388. doi: 10.1038/s41467-023-44173-3 (PMC10725445; doi:10.1038/s41467-023-44173-3)
Supplement: Supplementary file 1 — Supplementary Information [file 41467_2023_44173_MOESM1_ESM.pdf]

Supplementary Information

LinRace: cell division history reconstruction of single cells using paired lineage barcode and gene expression data

Xinhai Pan[1][0000-0002-3914-8489], Hechen Li[1][0000-0003-4907-429X], Pranav Putta[1][0000-0000-0000-0000], and Xiuwei Zhang[1][0000-0002-1713-772X]

Georgia Institute of Technology, Atlanta GA 30332, USA

Abstract. Lineage tracing technology using CRISPR/Cas9 genome editing has enabled simultaneous readouts of gene expressions and lineage barcodes in single cells, which allows for inference of cell lineage and cell types at the whole organism level. While most state-of-the-art methods for lineage reconstruction utilize only the lineage barcode data, methods that incorporate gene expressions are emerging. Effectively incorporating the gene expression data requires a reasonable model of how gene expression data changes along generations of divisions. Here, we present LinRace (**Lin**eage **R**econstruction with **a**symmetric **ce**ll division model), which integrates lineage barcode and gene expression data using asymmetric cell division model and infers cell lineages and ancestral cell states using Neighbor-Joining and maximum-likelihood heuristics. On both simulated and real data, LinRace outputs more accurate cell division trees than existing methods. With inferred ancestral states, LinRace can also show how a progenitor cell generates a large population of cells with various functionalities. LinRace is available at: https://github.com/ZhangLabGT/LinRace.

Keywords: CRISPR/Cas9 genome editing · single cell lineage reconstruction · Neighbor Joining · Maximum likelihood.

Table of Contents

1 Supplementary Figures 3
2 Supplementary Note 1 16
3 Supplementary Note 2 17
3.1 Experimental details of benchmarks on TedSim simulated datasets 17
3.2 Experimental details of benchmarks on real C.elegans dataset 17
3.3 Experimental details of lineage reconstruction of scGESTAULT dataset 18
3.4 Experimental details of running time comparisons 19

1 Supplementary Figures

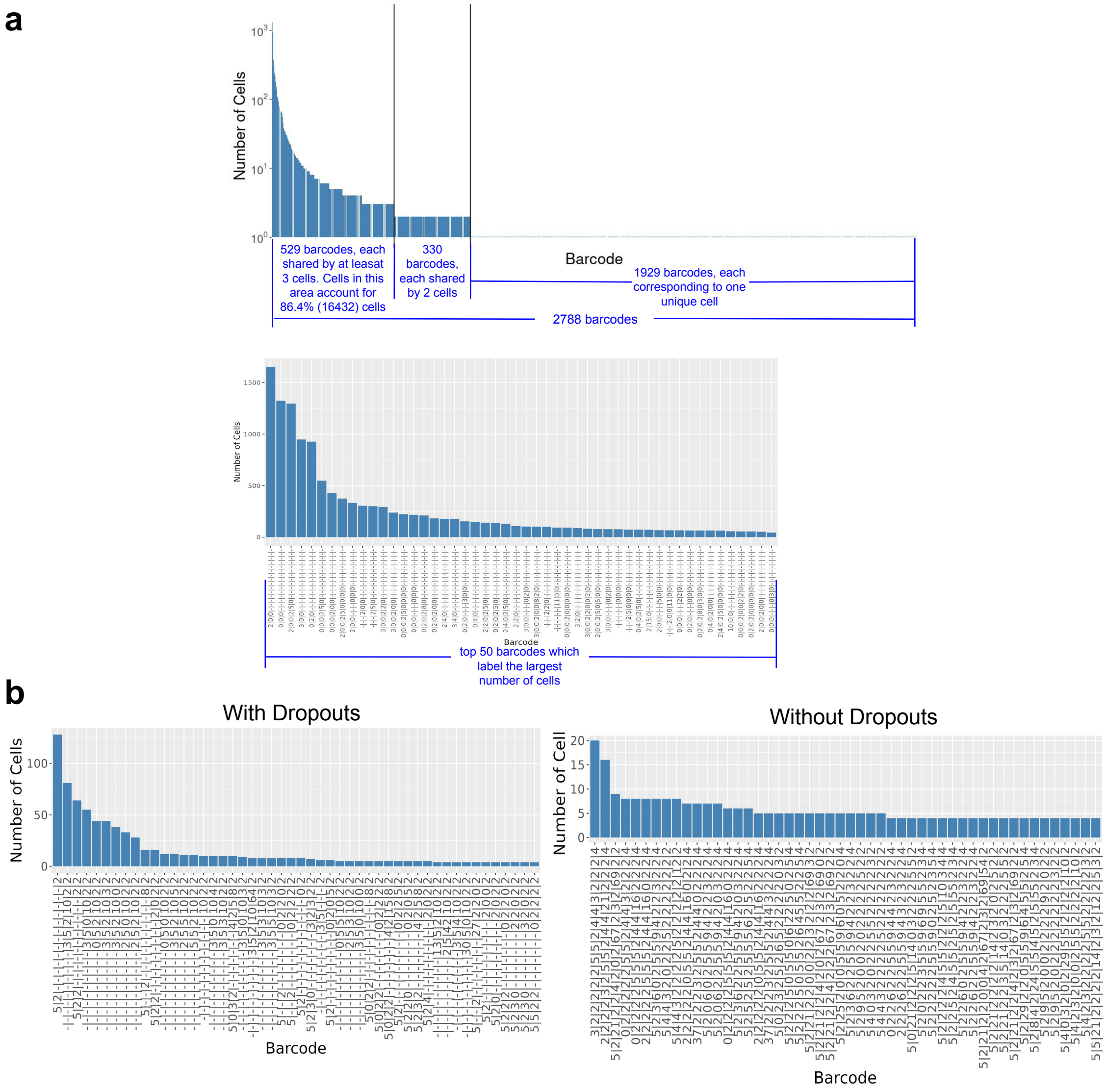

Supplementary Figure 1: Barcode distributions in both real and TedSim simulated datasets. x-axis shows barcode in the form of character strings, where “0” denotes unmutated state, nonzero numbers denote mutations, and “-” denotes dropouts. The y-axis shows the number of cells with the same barcode. **a** Barcode frequencies of the embryo2 dataset in M. Chan *et al.* The dataset has 19019 cells, 18 targets, and a total of 2788 unique barcodes. Top: the barcode frequencies of all barcodes; Bottom: the barcode frequencies of the top 50 barcodes with the highest frequencies. **b** Barcode frequencies of TedSim simulated datasets, one with dropouts and one without. Both datasets have 1024 cells, 16 targets for the barcode, and mutation rate = 0.1. Both plots show top 50 most frequent barcodes. Source data is provided as a Source data file.

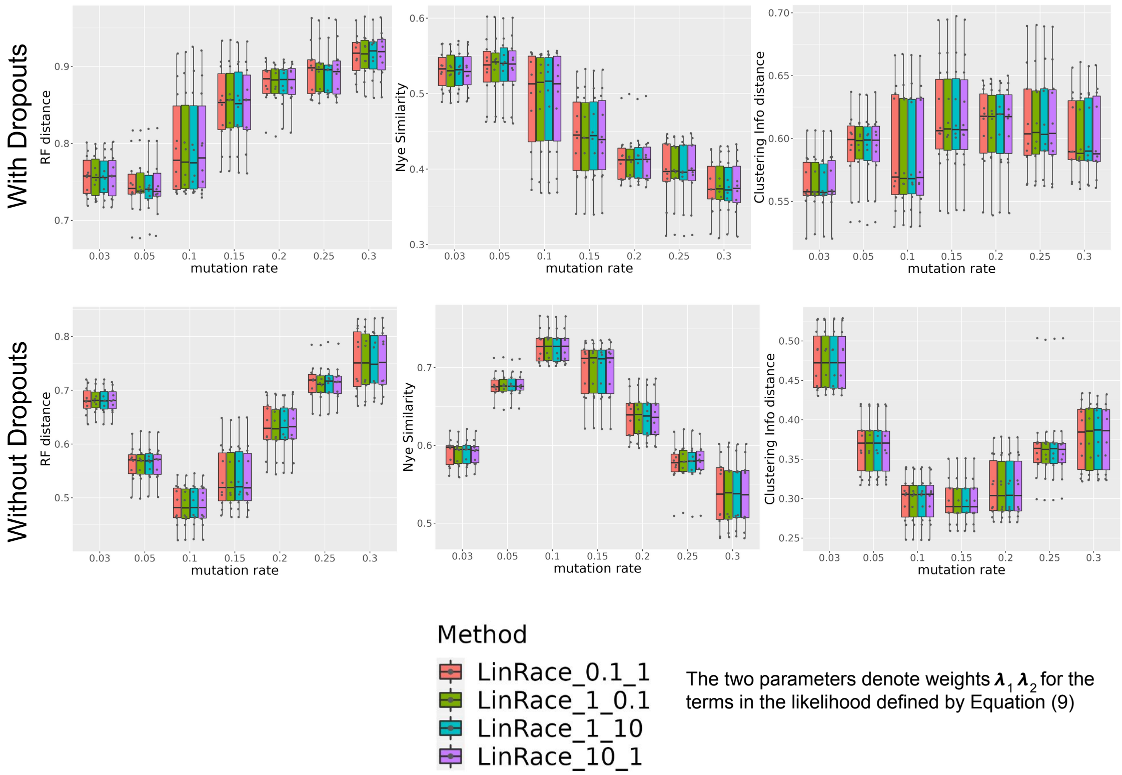

Supplementary Figure 2: Comparisons of LinRace with different settings of hyperparameters. Each color corresponds to a hyperparameter configuration. The color legend is in the format of LinRace_λ_1_λ_2, where the first parameter λ_1 is the weight for asymmetric division likelihood, and the second parameter λ_2 is the weight for neighbor distance likelihood. The datasets used are the same data used for benchmarking the lineage reconstruction methods in Fig. 2a. Source data are provided as a Source Data file.

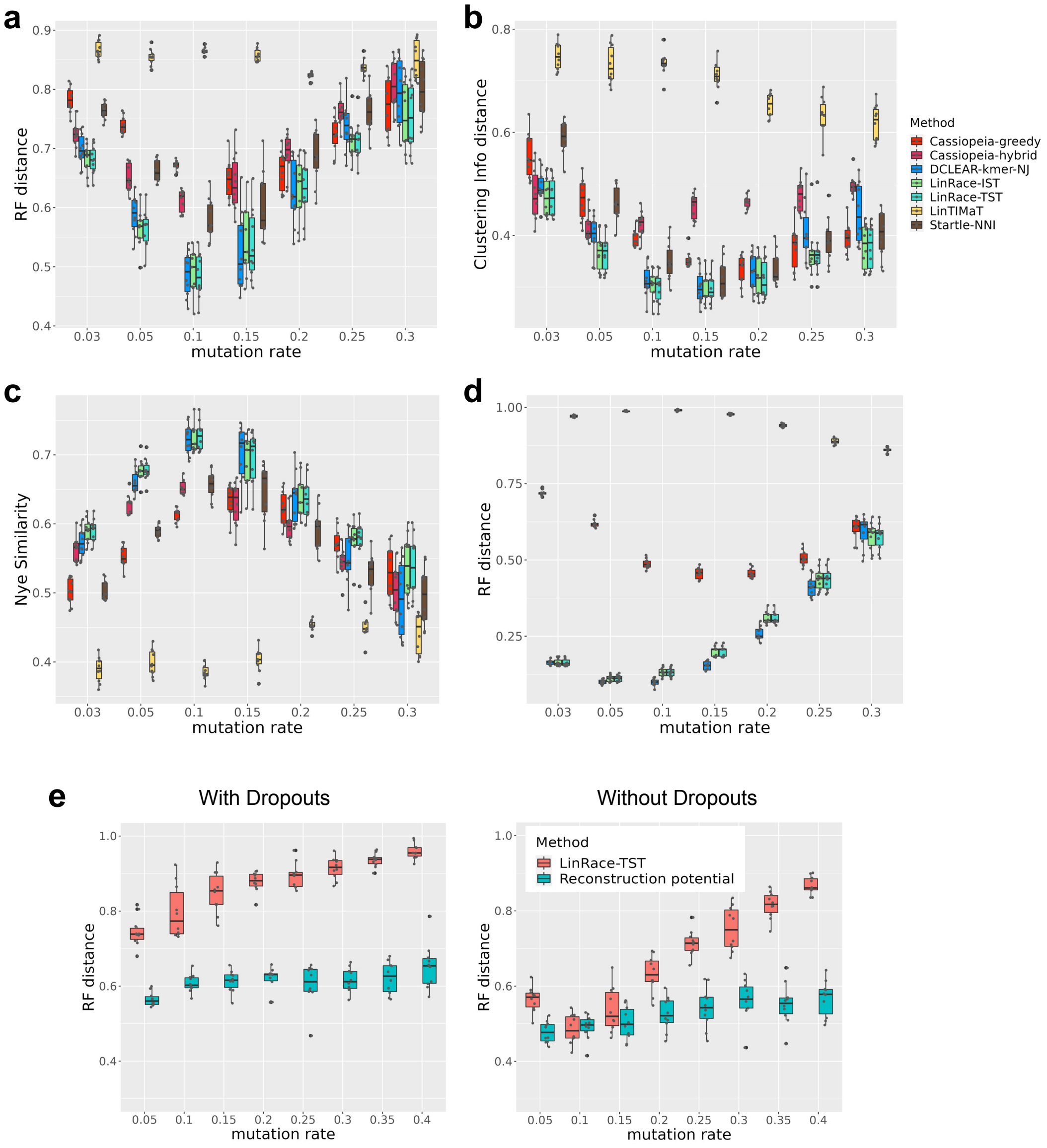

Supplementary Figure 3: Comparisons of LinRace (LinRace-IST and LinRace-TST) and other methods on TedSim simulated datasets without dropouts. The number of target sites is set to 64. The other simulation settings for gene expression data and parameters for running the algorithms are the same as described in Methods and Supplementary Note 2 for 1024 cells. For every combination of parameters, 10 simulated datasets are generated. **a** RF distance on datasets with 1024 cells. **b** Nye similarity on datasets with 1024 cells. **c** CID on datasets with 1024 cells. **d** RF distance on datasets with 4096 cells. Nye similarity and CID all have the range of $[0, 1]$. For both RF distance and CID, lower is better, and for Nye similarity, higher values indicate better performance. Only RF distance results are given on 4096 cells because Nye similarity and CID are too slow to run on such large datasets. Moreover, Startle-NNI is not included in the comparisons of 4096 datasets due to running time issues. **e** Comparison of the reconstruction potential and LinRace-TST performances. The data used to calculate the reconstruction potential and the RF distance of LinRace-TST reconstructed trees are the same data from Fig. 2 a-c. Source data are provided as a Source Data file.

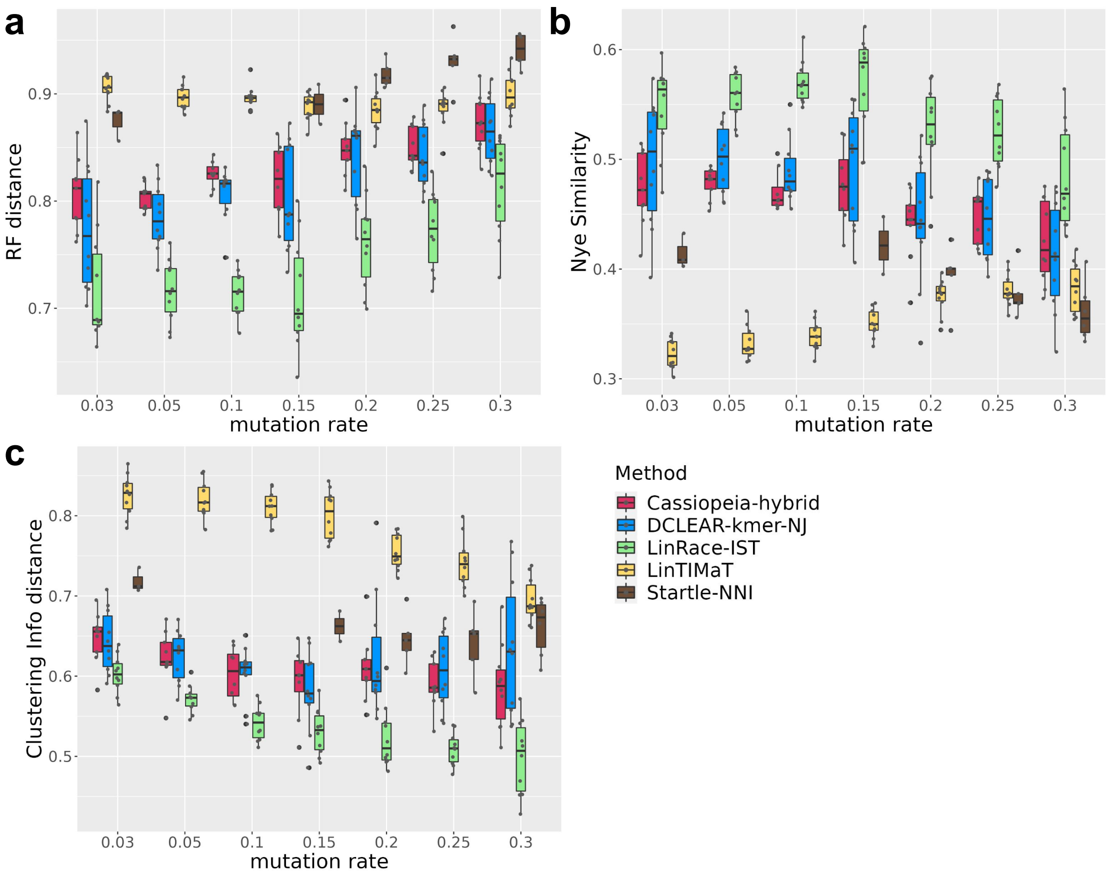

Supplementary Figure 4: Comparisons of LinRace (LinRace-IST) and other methods on TedSim simulated datasets of 1024 cells with dropouts. The number of target sites is set to 128. The other simulation settings for gene expression data and parameters for running the algorithms are the same as described in Methods and Supplementary Note 2. For every combination of parameters, 10 simulated datasets are generated. **a** RF distance comparisons. **b** Nye similarity comparisons. **c** CID comparisons. RF distance, Nye similarity, and CID all have the range of $[0, 1]$. For both RF distance and CID, lower is better, and for Nye similarity, higher values indicate better performance. Moreover, Some runs of Startle-NNI ($\mu = 0.05, 0.1$) are not available due to running time issues. Source data are provided as a Source Data file.

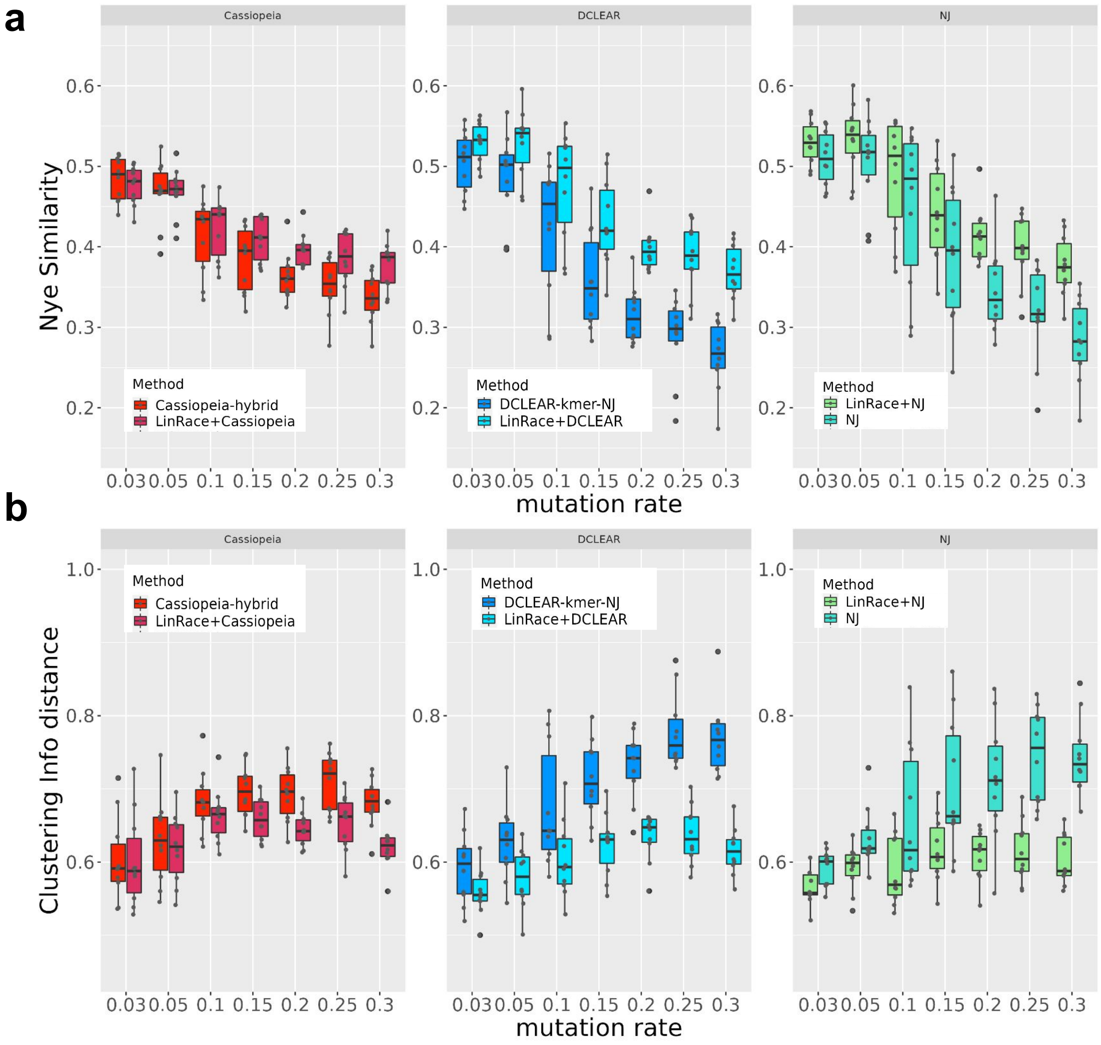

Supplementary Figure 5: Nye Similarity and CID Comparisons of LinRace's improvements upon different methods for building tree backbone including NJ, Cassiopeia-hybrid, and DCLEAR-kmer. In these plots, we used the same datasets as in Fig. 2 **a-c**. Source data are provided as a Source Data file.

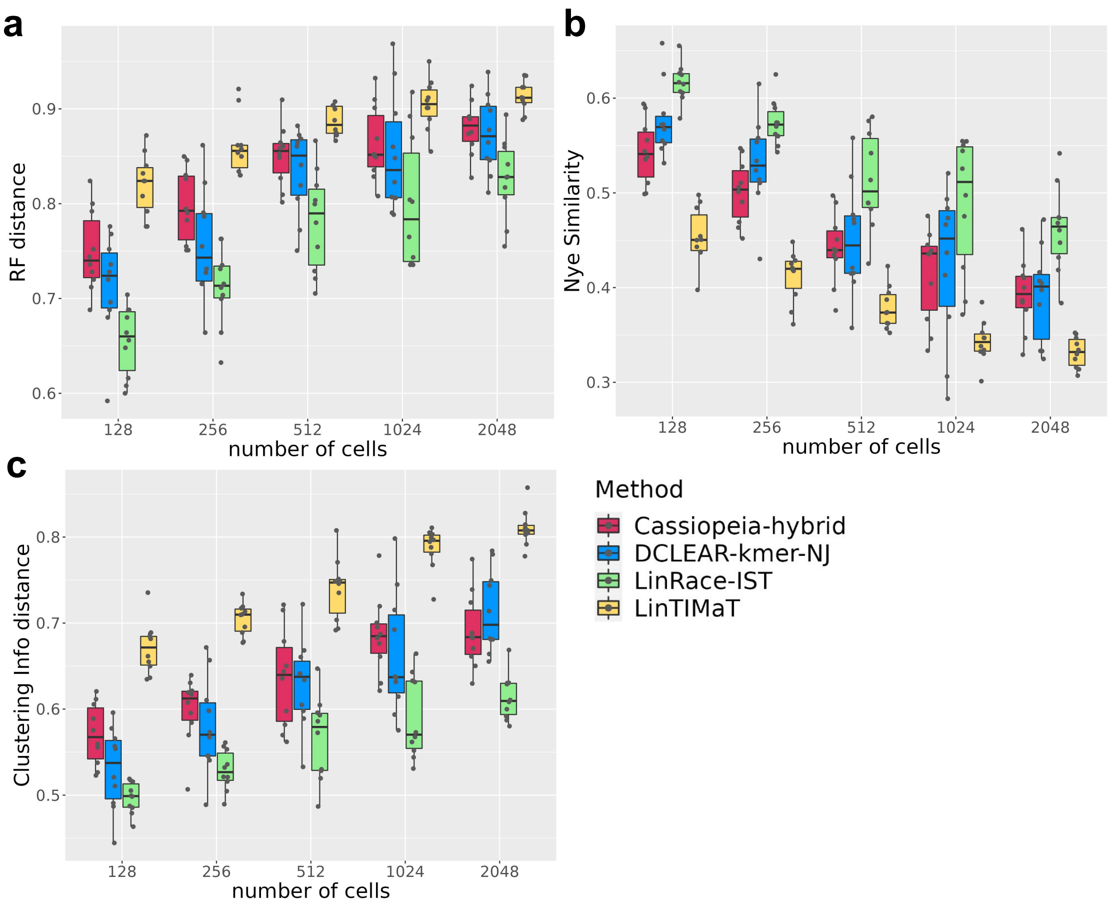

Supplementary Figure 6: Comparisons of LinRace and selected state-of-the-art methods on TedSim simulated datasets of varying numbers of cells with dropouts. The mutation rate is set to 0.1 and the number of target sites is set to 16. The other simulation settings for gene expression data and parameters for running the algorithms are the same as described in Methods and Supplementary Note 2. **a** RF distance comparisons. **b** Nye similarity comparisons. **c** CID comparisons. RF distance, Nye similarity, and CID all have the range of $[0, 1]$. For both RF distance and CID, lower is better, and for Nye similarity, higher values indicate better performance. Source data are provided as a Source Data file.

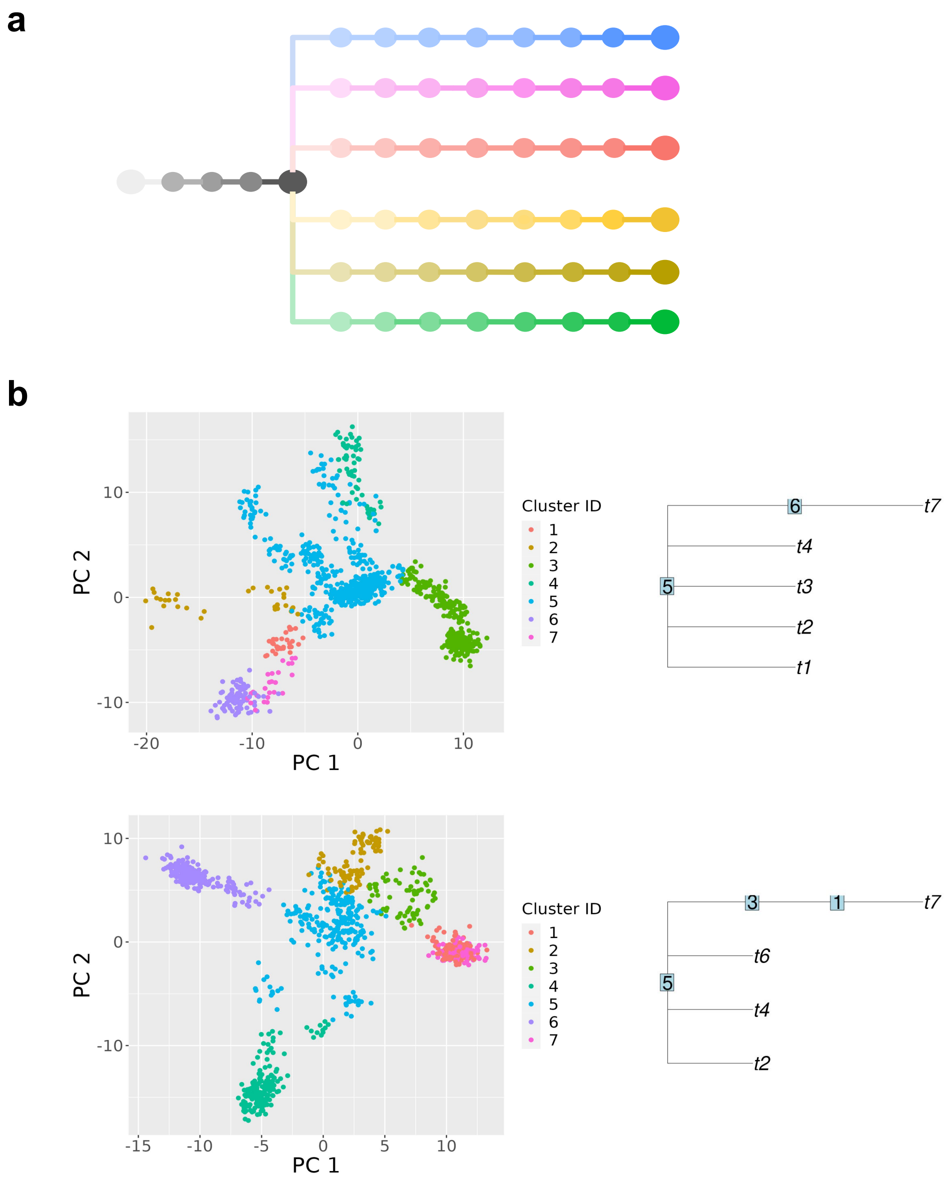

Supplementary Figure 7: **a** Ground truth cell state tree with sampled intermediate states ($step\_size = 0.5$). A total of 52 discrete states are sampled from the cell state tree. **b** 2-d PCA visualizations of TedSim simulated gene expression data and inferred cell state trees. Two examples here where on the left are the gene expressions; on the right are the inferred cell state trees using Slingshot. A random cell from the root cell type is given to Slingshot to guarantee the correct starting cell cluster.

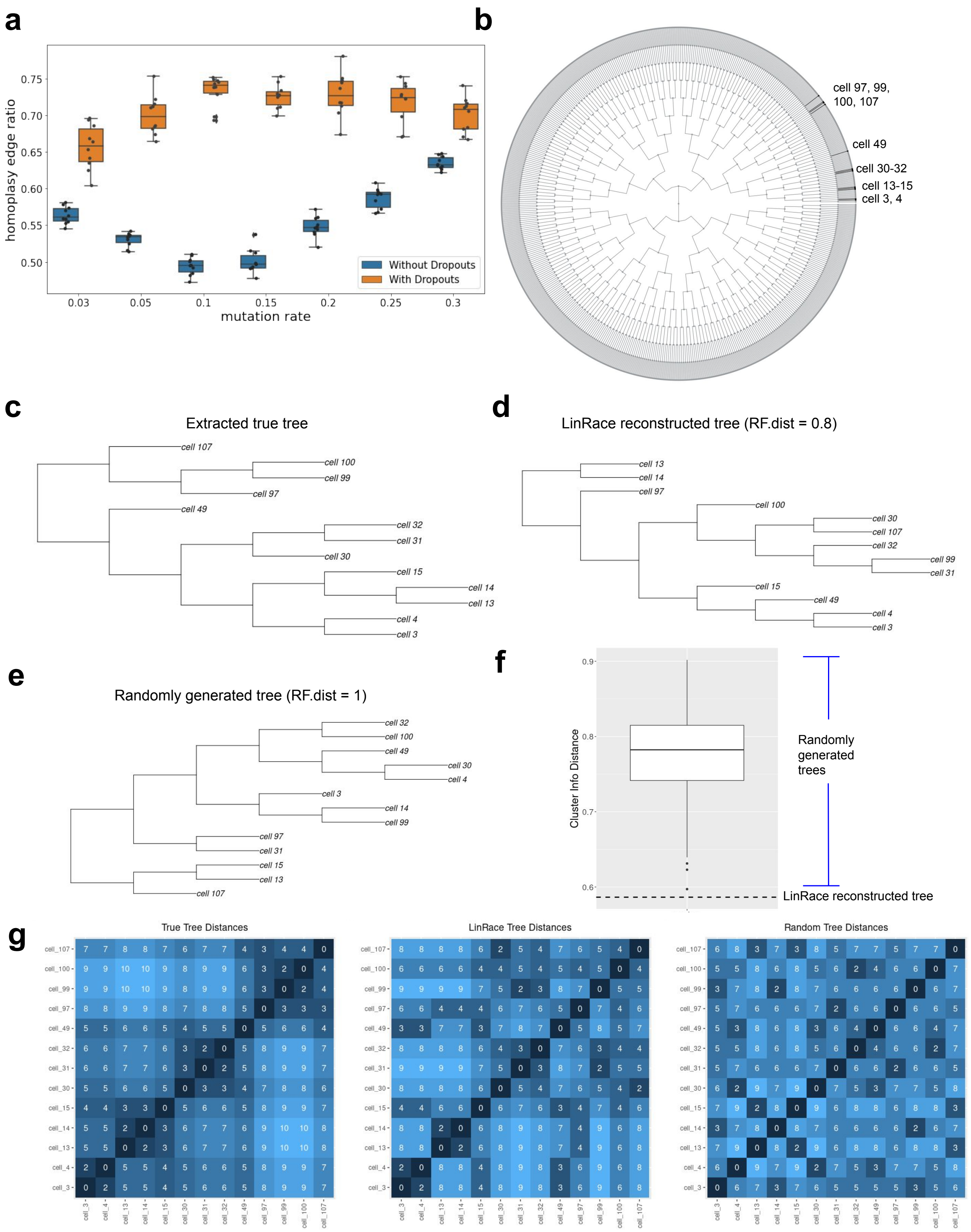
a
homoplasy edge ratio
mutation rate
Without Dropouts
With Dropouts
b
cell 97, 99, 100, 107
cell 49
cell 30-32
cell 13-15
cell 3, 4
c
Extracted true tree
d
LinRace reconstructed tree (RF.dist = 0.8)
e
Randomly generated tree (RF.dist = 1)
f
Cluster Info Distance
Randomly generated trees
LinRace reconstructed tree
g
True Tree Distances
LinRace Tree Distances
Random Tree Distances

Supplementary Figure 8: Barcode homoplasy analysis of LinRace on TedSim simulated datasets. Source data are provided as a Source Data file. **a** Barcode homoplasy edge ratio of TedSim generated datasets. We calculated the homoplasy edge ratio (as defined in Methods) on datasets with 1024 cells, which has the range of $[0, 1]$. Lower values indicate less barcode homoplasy and higher values indicate more barcode homoplasy. **b** Barcode homoplasy on the true lineage tree. Cells colored in black are the leaf cells sharing the same barcode. **c** Extracted true lineage tree of the homoplasic cells. The subtree is constructed by removing all other leaf cells and internal nodes and collapsing the edges connecting these cells together while maintaining the relative distances between the cells. The edge lengths are normalized to one. **d** The LinRace reconstructed tree of the homoplasic cells. The LinRace tree is constructed using LinRace's likelihood and local search framework. The RF distance between the LinRace reconstructed tree and the extracted true tree shown in **c** is 0.8. **e** One example of randomly generated trees of the homoplasic cells. The randomly generated trees are constructed using ape's rtree function which generates binary trees of the input leaf cells. The RF distance between the randomly generated tree and the extracted true tree shown in **c** is 1. **f** Cluster Info Distances between the extracted true tree and randomly generated tree/LinRace reconstructed lineage. **g** Pairwise graph distances of the true tree, LinRace tree, and a random tree. The heatmaps reflect the exact tree topology shown respectively in **b,e,f**.

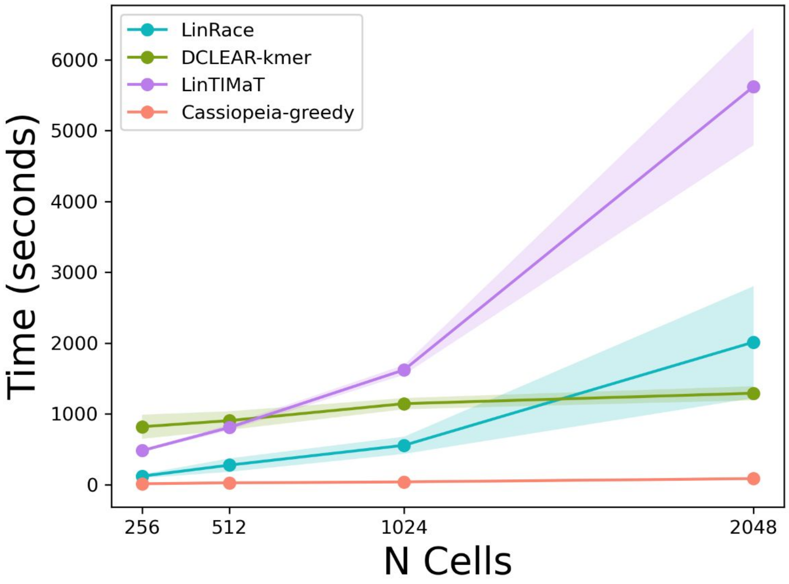

Supplementary Figure 9: Comparisons of running time of lineage reconstruction methods. The mutation rate is set to 0.1 for all datasets in this test. The detailed descriptions for simulation and method settings can be found in Supplementary Note 2. Source data are provided as a Source Data file.

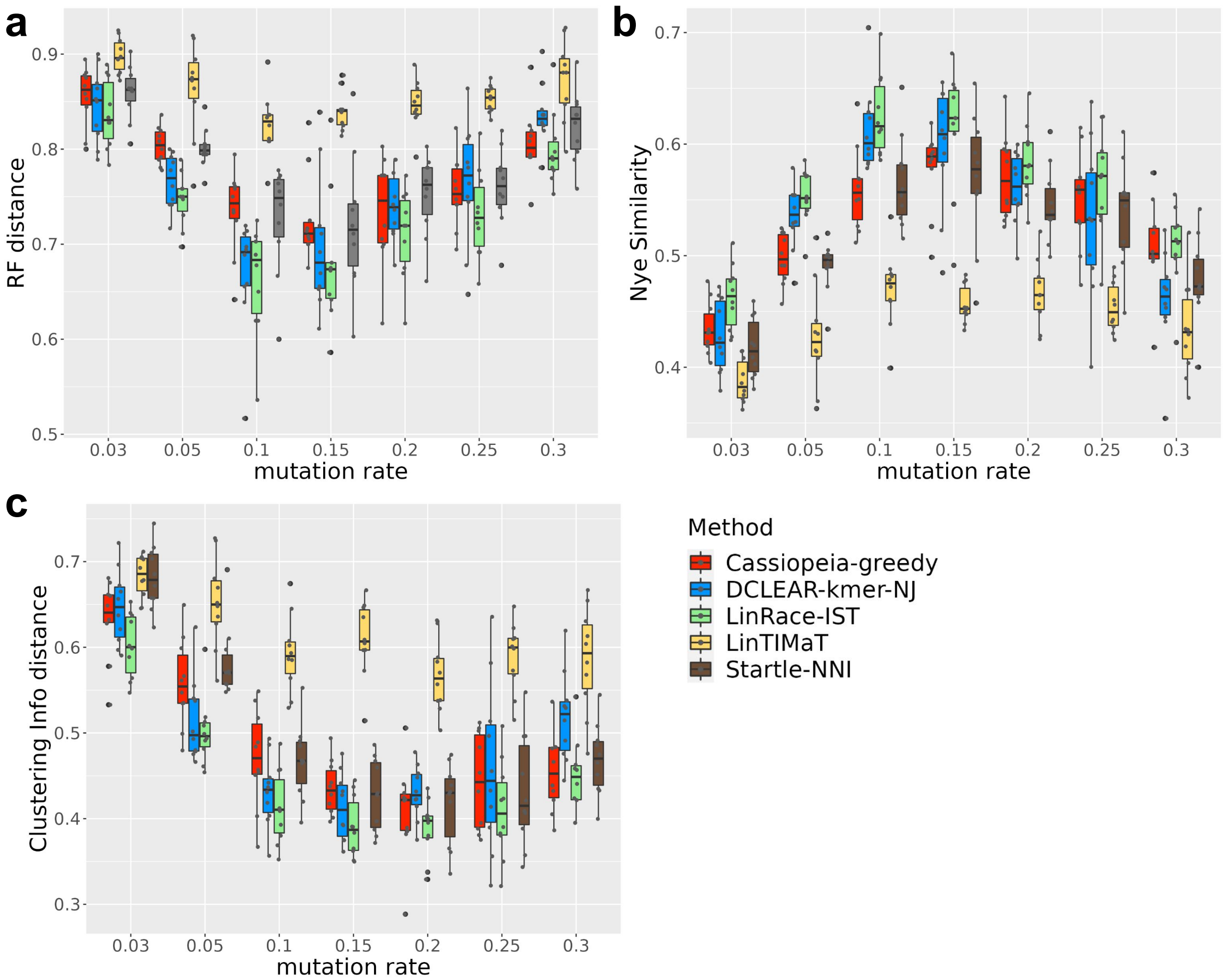

Supplementary Figure 10: Benchmarking result on the *C. elegans* dataset with simulated barcodes. The methods are tested for varying mutation rates without dropouts. Three metrics, RF distance, Nye similarity, and CID are used for the benchmark. For every combination of parameters, 10 simulated datasets are generated. **a** RF distance comparisons. **b** Nye similarity comparisons. **c** CID comparisons. RF distance, Nye similarity and CID all have the range of [0, 1]. For both RF distance and CID, lower is better, and for Nye similarity, higher values indicate better performance. Source data are provided as a Source Data file.

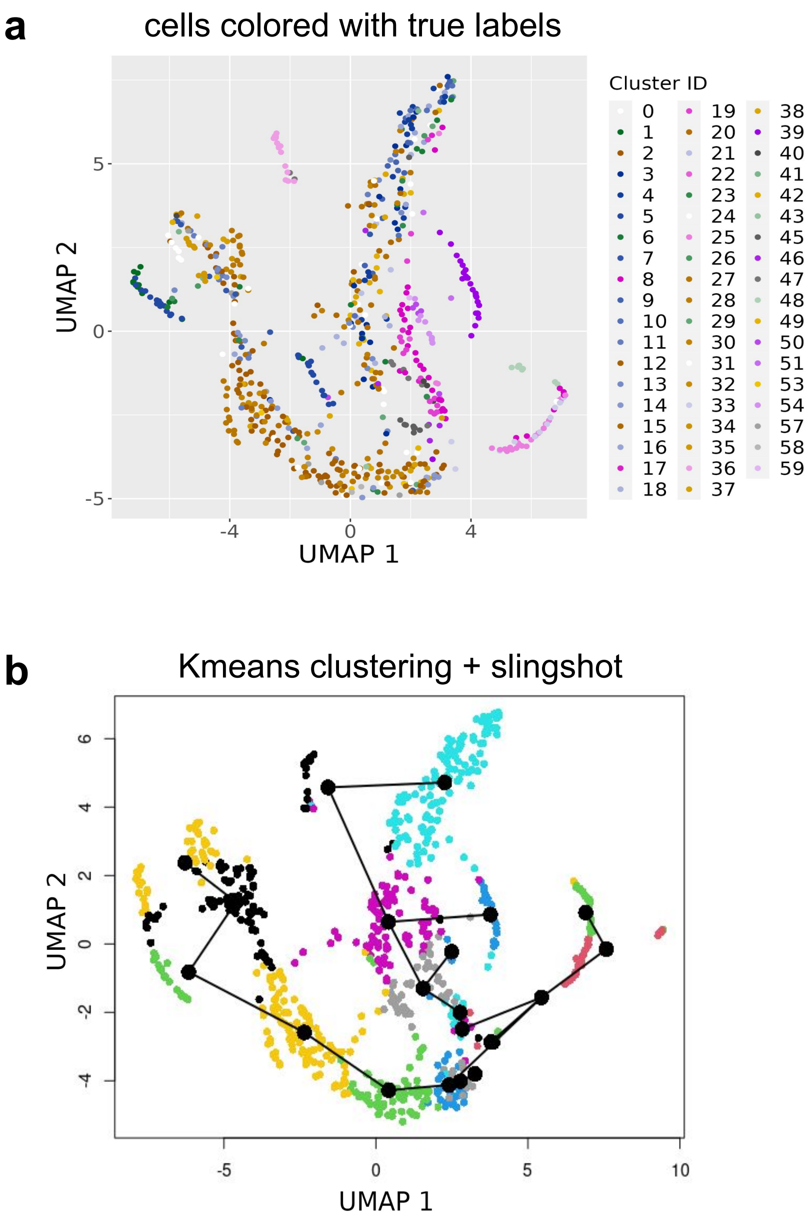

Supplementary Figure 11: **a** UMAP visualization of scGESTAULT datasets. The true labels are annotated labels from the original paper and the color code is consistent with **Fig. 4** in the main manuscript. **b** For LinRace, we used $k$means + Slingshot fitted cell states and trajectories to calculate the likelihood. For $k$means, we set $k = 20$ and for Slingshot we used the first 20 PCs and did not provide information about the root cell state. Source data are provided as a Source Data file.

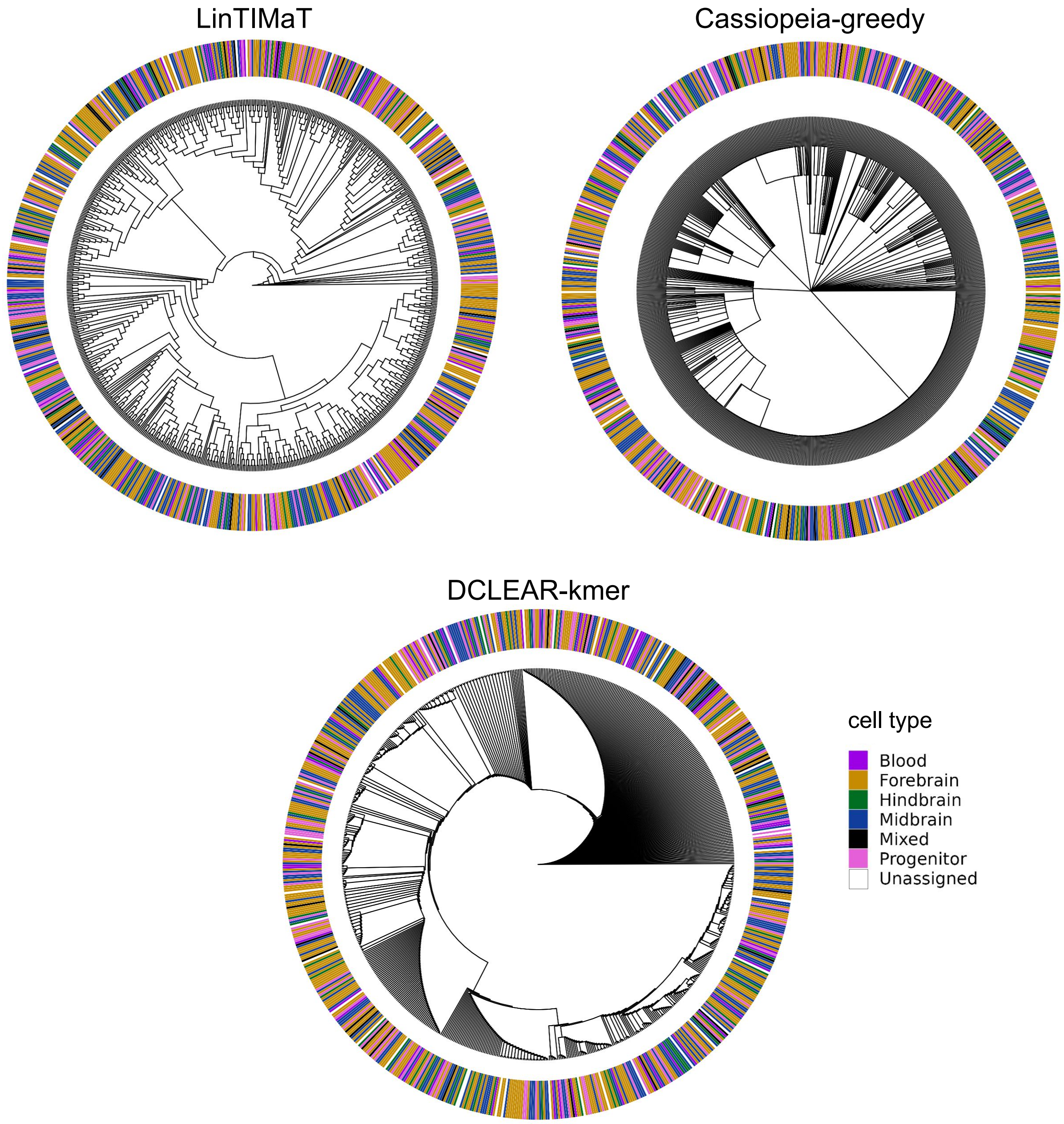

Supplementary Figure 12: Reconstructed lineage trees of the scGESTAULT dataset using LinTIMaT, Cassiopeia-greedy, and DCLEAR-kmer. The color assignments on the outer ring show cell-type labels from the original paper. Source data are provided as a Source Data file.

2 Supplementary Note 1

The pseudocode of GES local search using rSS is given below.

Algorithm 1 GES local search using rSS

input gene expression data X
output T_{cell},CIV,S,M
Initialize GES as a random bifurcating tree $T_{cell} \leftarrow \textsc{BifurTree}(X)$
Infer cell states S and cell state trajectories T_{state} from X
Infer Ancestral states on the candidate tree $S \leftarrow \textsc{AncesInfer}(S,T_{cell})$
Initialize max likelihood as the score of the current tree $l_{max} \leftarrow \textsc{LikelihoodCal}(T_{cell})$
while $i \leq maxIter$, **do**
    Propose a new tree using rSS: $T_{new} \leftarrow \textsc{rSS}(T_{cell})$
    Infer Ancestral states on the new tree $S \leftarrow \textsc{AncesInfer}(S,T_{new})$
    Calculate likelihood on the new proposed tree $l \leftarrow \textsc{LikelihoodCal}(T_{new})$
    if $l > l_{max}$ **then**
        Move to the new tree: $T_{cell} = T_{new}$, $l_{max} = l$
    end if
    if l_{max} converged **then**
        Restart with a new random bifurcating tree $T_{cell} \leftarrow \textsc{BifurTree}(X)$
    end if
    $i++$
end while

3 Supplementary Note 2

3.1 Experimental details of benchmarks on TedSim simulated datasets

(1) Synthetic cell state tree used in the TedSim benchmarks is given in Fig. 1. The edge lengths of leaf states are set to 4 and the root edge is set to 2.

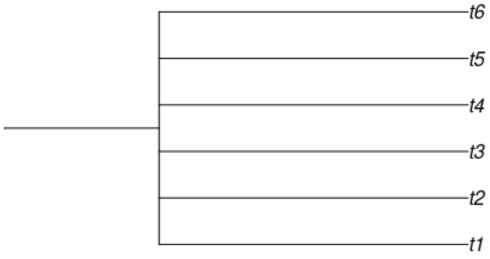

Figure 1: A synthetic state tree with 6 branches is used to benchmark the lineage reconstruction methods.

On the other hand, for LinRace-IST, we use Slingshot to infer the cell state tree. Moreover, we select a random cell ID from the root cell cluster as the starting cell for Slingshot which will guarantee the correct starting cell type for the inferred cell state tree.
(2) Variables:

a) Mutation rate: $\mu = [0.03, 0.05, 0.1, 0.15, 0.2, 0.25, 0.3]$.
b) Number of cells(ncells): 1024 or 4096.
c) Dropout: $p_d = 0$ or 1.
d) Number of target sites(Nchar): For 1024 cells, $Nchar = 16, 128$; for 4096 cells, $Nchar = 64$.

We run 10 instances for each combination of the variables.
(3) Other Simulation parameters:

Number of genes (ngenes)	500
Number of Identity Vectors (N_{IV})	30
State Identity Vector stepsize ($step$)	0.5
Maximum number of state shifts for one division (max_walk)	5
Identity Vector center (starting value for diff-IF)	1
Number of diff-Identity Vectors (N_{diff})	20
nondiff-SIV standard deviation (σ)	0.5
Probability of nonzero gene effect (ge_prob)	0.3
Probability of outlier gene (prob_hge)	0.03
Mean of capture efficiency α (alpha_mean)	0.1
Standard deviation of capture efficiency α (alpha_sd)	0.1

3.2 Experimental details of benchmarks on real C.elegans dataset

(1) Inferred cell state tree using Slingshot:
The edge lengths are set to 2. Moreover, we select a random cell ID from a specific cell cluster as the starting cell that achieves a relatively balanced cell state tree.
(2) Variables:

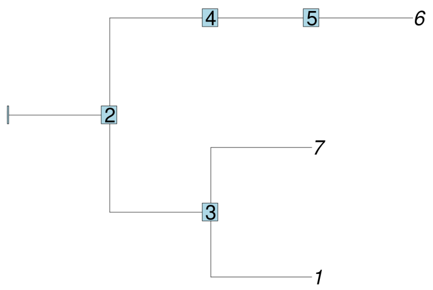

Figure 2: The cell state tree of C. elegans inferred using Slingshot.

Mutation rate: μ = [0.05,0.1,0.15,0.2,0.25,0.3,0.35,0.4].
Dropout: $p_d = 0$ or 1.
Distribution of mutated states: $unif_{on} = 0$ or 1.
We run 10 instances for each combination of the variables.
(3) Other Simulation parameters:

Number of cells (ncells)	363
Number of target sites (Nchar)	9

(4) Lineage reconstruction methods settings:

– LinRace:
  - max number of iteration for each local search: 500,
  - Weight for asymmetric division likelihood: $\lambda_1 = 10$,
  - Weight for neighbor distance likelihood: $\lambda_2 = 1$
  - We use kmeans to infer the cell clusters of the first 20 PCs of the data where $k = 7$. Then, we use Slingshot to infer the state trajectories and finally transform it into a cell state tree as an input to LinRace.

– Startle-NNI:
  - iterations: 5000
  - number of threads: 8

– LinTIMaT:
  - number of genes: -gc 93,
  - number of mutation likelihood iterations: -mi 20000,
  - number of combined likelihood iterations: -ci 20000.

– DCLEAR-kmer: k-mer length k = 2

– Cassiopeia-greedy: No prior knowledge is used for the probability of induced characters.

3.3 Experimental details of lineage reconstruction of scGESTAULT dataset

We use the ZF1_F3 sample from the scGESTAULT dataset which contains 750 cells and 60 cell states. The 60 cell states are annotated by the original paper and are classified into Forebrain, Hindbrain, Midbrain, Blood, Progenitor, Mixed, and Unassigned cell types. For LinRace, we use a predefined state lineage structure where the "progenitor" cell type is the root cell state, and can lead to all the other annotated cell types including "Forebrain", "Midbrain", "Hindbrain", "Blood" and "Mixed". The settings for running the lineage reconstruction methods are shown below:

– LinRace:

    - max number of iteration for each local search: 500,
    - Weight for asymmetric division likelihood: $\lambda_1 = 10$,
    - Weight for neighbor distance likelihood: $\lambda_2 = 1$
    - We use kmeans to infer the cell clusters of the first 20 PCs of the data where $k = 7$. Then, we use Slingshot to infer the state trajectories and finally transform it into a cell state tree as an input to LinRace.
- Startle-NNI:
    - iterations: 5000
    - number of threads: 8
- LinTIMaT:
    - number of genes: -gc 100,
    - number of mutation likelihood iterations: -mi 20000,
    - number of combined likelihood iterations: -ci 20000.
- DCLEAR-kmer: k-mer length k = 2
- Cassiopeia-greedy: No prior knowledge is used for the probability of induced characters.

3.4 Experimental details of running time comparisons

(1) Dynamically determine the max number of iterations for local search in LinRace:

(2) Lineage reconstruction methods settings:

- LinRace:
    - max number of iteration for each local search: 300,
    - Weight for asymmetric division likelihood: $\lambda_1 = 10$,
    - Weight for neighbor distance likelihood: $\lambda_2 = 1$
    - Asymmetric division rate: $p_a = 0.8$
- LinTIMaT:
    - number of genes: -gc 100,
    - number of mutation likelihood iterations: -mi 25000,
    - number of combined likelihood iterations: -ci 25000.
- DCLEAR-kmer: k-mer length k = 2
- Cassiopeia-greedy: No prior knowledge is used for the probability of induced characters.

(3) Simulation parameters for gene expression data are kept the same as **Sec. 2.2**.
